# EULAR 2023 Recommendations for the Management of Systemic Lupus Erythematosus: One Step Forward

**DOI:** 10.31138/mjr.130124.erm

**Published:** 2024-03-31

**Authors:** Georgia-Savina Moysidou, Antonis Fanouriakis

**Affiliations:** Rheumatology and Clinical Immunology Unit, ‘Attikon’ University Hospital, Athens, Greece

**Keywords:** recommendations, systemic lupus erythematosus

The European Alliance of Associations for Rheumatology (EULAR) recommendations for the management of systemic lupus erythematosus (SLE) have become a ‘tradition’ since their first publication in 2008, guiding physicians who care after patients with this challenging disease. In their most recent update in 2023, the respective first Task Force was expanded to include, for the first time, renowned lupus experts from four continents, in an effort to reflect practice all around the globe. To facilitate dissemination of the recommendations, the latter were shortened compared to previous versions, counting a total of 13 recommendations. Highlights include further reductions in the recommended maintenance dose of glucocorticoids (GC), a strong recommendation for early use of conventional and biologic immunosuppressive drugs and the use of combination therapies in lupus nephritis (LN).

## GLUCOCORTICOIDS: ENVISIONING THEIR USE AS BRIDGING THERAPY

GC along with hydroxychloroquine remain one of the cornerstones of the treatment of SLE, exerting their effects through genomic and non-genomic pathways.^1^ Dose and route of administration varies depending on prevailing disease manifestations and their severity. Daily dose is categorised as low (≤ 7.5 mg/day of prednisone-equivalent), medium (7.5–30 mg/day), high (30–100 mg/day) and very high (>100mg), but the latter are almost never used nowadays.^2^ GC may provide rapid symptom relief, however prolonged treatment is associated with an increased risk for infections, metabolic disturbances such as diabetes mellitus, cardiovascular events, irreversible organ damage and mortality.^3,4^ To this end, the target is GC minimisation and, if feasible, complete withdrawal. This recommendation is not limited to SLE, but the latter has proven notoriously difficult to be treated with GC-free regimens, and a subset of patients are dependent on GC for symptom control. In the 2019 recommendations, the ‘acceptable’ threshold of daily prednisone dose for maintenance treatment was ≤ 7.5 mg/day prednisone equivalent^5^; this was changed to 5 mg/day in the current update,^6^ in a statement that received 96.3% agreement and mean (SD) level of agreement 9.57 (0.77).

It should be noted that this recommendation was not based on randomised data (as was the case for example in ANCA-associated vasculitides, where two different GC regimens were also tested during the PEXIVAS trial^7^; rather, observational studies indicate that in a setting of low disease activity, a dose ≤ 5 mg/day prednisone equivalent is associated with lower damage accrual as compared to a dose ≤ 7.5 mg/day.^8^ To facilitate faster GC tapering, in moderate/severe disease the use of GC pulses (250–1000 mg/day for 1–3 days) should be considered.^6^ Of note, low dose pulses regimens (1500mg in three days) are equally effective as high dose pulses (3000–5000mg).^9^ Treatment with pulse GC may be followed by per os prednisone at doses of 0.3–0.5 mg/Kg/day with tapering; the use of the traditional 1 mg/kg regimen does not lead to an additional therapeutic benefit, given that the genomic pathway is nearly completely saturated at doses >30mg prednisone-equivalent per day.^10^

Complete GC withdrawal remains the ultimate treatment target. However, it is frequently challenging in clinical practice due to the potential risk for flare. In the CORTICOLUP open-label randomised trial in 124 SLE patients with sustained remission, GC maintenance (on 5mg/day) was superior to their abrupt discontinuation in terms of time to first flare, occurrence of mild/moderate flares and moderate to severe flares^11^; despite this setback, observational studies suggest that GC discontinuation can be feasible following slow tapering in SLE patients with prolonged remission.^12^ With the hope that new drugs will enrich our armamentarium in the future, it is the vision of the Task Force that GC will be considered as a bridging therapy in lupus, in a treatment paradigm similar to rheumatoid arthritis.

## CONVENTIONAL IMMUNOSUPPRESSIVES AND BIOLOGICS: AS EARLY AS NEEDED

Another major issue addressed in the 2023 update was the advocacy for an early use of immunosuppressive drugs (IS) and biological agents. Since the previous recommendations, anifrolumab, a human monoclonal antibody targeting the type I interferon receptor subunit 1, has been approved for SLE.^13^ The expanding therapeutic options and ambition to ‘do away with prednisone’ support the addition of immunomodulating/immunosuppressive agents and/or biological agents (i.e belimumab or anifrolumab) in any patient who does not respond to hydroxychloroquine or is unable to reduce GC below 5 mg/day. There is growing evidence that early use of IS agents is protective against relapses in patients with severe haematological SLE and immune thrombocytopenia.^14,15^

Importantly, no hierarchy is given between IS or biologic therapies; in other words, biologics need not be considered only after failure to one or more conventional synthetic drugs. Although this issue is still a matter of debate, mainly due to the high cost of biologics, it cannot be disputed that belimumab and anifrolumab have proven significant efficacy and safety in SLE patients in large high-quality RCTs,^13,16,17^ an accomplishment lacking for all synthetic immunosuppressives, the use of which has been largely based on physician experience. Indeed, belimumab leads to a reduction of disease flares, organ damage accrual and has a significant GC sparing effect, shown in the BLISS trials and corroborated now by multiple real-life observational studies.^18–22^ Anifrolumab, on the other hand, showed superiority over standard-of-care in the BICLA and almost all secondary endpoints in the phase 3 TULIP trials, including a GC-sparing effect^23^; real-world data on its long-term efficacy are still scarce,^24^ although the drug seems to demonstrate high efficacy in cutaneous manifestations in preliminary reports.^25^ Notably, the safety profile of biologic drugs is also probably more favourable than most synthetic immunosuppressives, as the latter are frequently complicated by side-effects (gastrointestinal toxicity, hair loss, liver dysfunction).

Based on the above, the EULAR recommendations aimed not to be unfair to biologic drugs, only based on their significantly higher cost. Nevertheless, as stated in the discussion of the manuscript,^6^ the Task Force recognised that ‘for the majority cases it may be prudent to try at least one conventional immunosuppressive’ prior to a biologic agent, to take into account the variable access to biologics in various parts of the world.

## LUPUS NEPHRITIS: UPFRONT COMBINATION TREATMENTS TO BE CONSIDERED, ALTHOUGH NOT MANDATORY

Although cyclophosphamide (CYC) and mycophenolate mofetil (MMF) remain the anchor drugs in the treatment of LN, the 2023 EULAR recommendations underscore the potential for combination therapies, even as a first-line treatment. This recommendation was based on the positive results of two large RCTs, the Belimumab International Study in Lupus Nephritis (BLISS-LN) and Aurinia Renal Response in Active Lupus With Voclosporin (AURORA-1),^22,26^ as well as post-hoc analyses of these trials. The wording ‘should be considered’ was chosen for both combination therapies, applicable to all patients with active LN.

The ‘verdict’ of the updated recommendations regarding the position of combination therapies in LN was long awaited, since there are arguments and counterarguments for a universal recommendation of initial triple therapy. Kidney involvement is by default a severe complication of SLE, with significant impact on long-term prognosis; moreover, rates of complete response with standard-of-care treatment remain frustratingly low in clinical trials. Most importantly, both belimumab and voclosporin proved superiority over established therapies for LN in large RCTs^22,26^ of two- and one-year duration, respectively. These points support advocacy for early combination therapy. On the other hand, it should be noted that belimumab and voclosporin did not perform equally well in all settings. Effects of belimumab were less profound when combined with CYC, while both drugs did not exhibit superiority over placebo in class V LN.^22^ Furthermore, a substantial proportion of patients responds to standard treatment in real-life settings (potential for overtreatment), while safety issues, higher cost, and possible limited access to these drugs in certain healthcare systems should also be taken into account. All these parameters have to be weighed in the final individualised decision, including patient preferences (shared decision making, a key overarching principle in SLE management). In the end of the day, irrespective of choice of therapy in every individual case, the most important step forward is that more treatment options are now available in LN. Regarding the duration of IS treatment, the EULAR update suggests continuation for at least 3 years, following clinical response.

## CONCLUSION

By definition, recommendations on the management of a disease cannot capture all aspects of everyday clinical practice, with its subtle ‘shades’ and -more than occasional- grey areas. Every patient is a different story and there is no ‘one size fit all’ approach. Acknowledging this reality, the 2023 update of the EULAR recommendations attempted to provide the state-of-the-art regarding the current treatment of SLE. Recent approval of novel drugs, validation of treatment targets and advances in treatment strategies justify the notion that this update constitutes a step forward in the management of this fascinating disease.
